# Adapting a community-based intervention to address social determinants of health influencing pre-exposure prophylaxis services for Black adults in Washington, District of Columbia: A study protocol

**DOI:** 10.1371/journal.pone.0290631

**Published:** 2023-11-03

**Authors:** Donaldson F. Conserve, Waimar Tun, DeMarc A. Hickson, Jennifer Gomez- Berrospi, Samuel Janson, Bukola Rinola, Mallory Durkin, Christian Buchanan, Christian Morris, Alia Saleeban, Kelia Olughu, Julie Pulerwitz, Deanna Kerrigan

**Affiliations:** 1 George Washington University, Milken Institute School of Public Health, Washington, DC, United States of America; 2 Population Council, Washington, DC, United States of America; 3 Us Helping US, People Into Living, Inc., Washington, DC, United States of America; 4 California State University, Fullerton, CA, United States of America; 5 Howard University, Washington, DC, United States of America; Kilimanjaro Clinical Research Institute, UNITED REPUBLIC OF TANZANIA

## Abstract

Community-based HIV treatment initiation and continuation helps to address social determinants of health (SDOH) barriers to care and increase antiretroviral therapy (ART) uptake and adherence. Similarly, community-based pre-exposure prophylaxis (cbPrEP) services can help address SDOH barriers such as transportation costs and stigma. However, few studies have examined cbPrEP programming in the Washington, District of Columbia (DC) area where more Blacks are disproportionately affected by HIV and have low PrEP uptake. This study aims to adapt and pilot a community-based ART intervention (cbART) intervention for cbPrEP service delivery for Black adults in the Washington, DC area. The adaptation of the cbART intervention will be informed by the ADAPT-ITT framework and the Consolidated Framework for Implementation Research. For Aim 1, in-depth and key informant interviews will be conducted with PrEP program managers at community-based organizations (N = 10), DC health department representatives (N = 8), PrEP providers (N = 10) and current and potential Black PrEP users (n = 24). The interviews will provide an initial *assessment* of barriers and facilitators to PrEP services and inform the *decisions* on how to *adapt* the cbART intervention for cbPrEP services. In Aim 2, we will train and pilot test the cbPrEP intervention for acceptability, feasibility, and appropriateness with Black adults (n = 60). Enrolled participants will complete a survey at baseline and at 45 days post-enrollment. In-depth interviews will be conducted with a subset (N = 16) of participants, those who did not enroll (N = 10) and providers implementing the cbPrEP intervention (N = 8). Alternative strategies to PrEP service delivery are needed to increase PrEP uptake among those most in need in the DC area. If cbPrEP delivery is found to be acceptable, feasible, and appropriate, it could have a significant impact on DC’s Ending the HIV Epidemic efforts and will inform future efforts to investigate the intervention’s efficacy on PrEP uptake and continuation among Black adults in DC.

## Introduction

The Centers for Disease Control and Prevention estimates that 1 in 13 residents in Washington, District of Columbia (DC) will acquire HIV in their lifetime. The DC Department of Health’s 2020 Annual Surveillance Report identified 282 new HIV cases diagnosed in 2019 [1]. Of those newly HIV diagnosed (2015–19), 1 in 4 were Black women and 2 in 5 were men who have sex with men of color (MSM) [1]. Black heterosexual men and women were 3.3 and 34.7 times more likely, respectively, to be living with HIV than their White counterparts [1]. Although HIV incidence is high, pre-exposure prophylaxis (PrEP) initiation is very low among Black adults in the DC area. As of 2019, there were approximately 4,000 PrEP users reported in DC [2] out of an estimated 14,000 PrEP users needed to reduce incidence by half [2, 3]. In Washington, DC, Whitman-Walker Health (a Federally Qualified Healthcare Center) is the largest PrEP prescriber in Washington DC (with over 2,500 PrEP prescriptions) and reports that 21% of its PrEP patients identify as Black [4]. In contrast, Blacks make up two-thirds of all new HIV diagnoses in Washington, DC [3].

The DC Department of Health (DOH) reports that in order to reduce HIV incidence by 56%, 13,392 DC residents that are most vulnerable for HIV acquisition would need to initiate PrEP [3, 5]. Yet, many barriers related to social determinants of health (SDOH) and other factors impede access to healthcare, including HIV and PrEP services, such as medical mistrust, low knowledge of PrEP, fear of potential side effects, community stigma, low knowledge of HIV services, negative interactions and anticipated stigma from healthcare providers, and transportation costs [6–11]. Community-based same-day HIV treatment initiation interventions has helped to address many of the aforementioned SDOH for HIV PrEP service access barriers [12–14]. Additionally, Black urban young adults have reported a preference to access PrEP services near their homes or near locations that are accessible by public transportation to overcome transportation barriers [7].

Evidence from low-and-middle-income countries, including from our own study [15], indicates that community-based (i.e. home, tent, mobile clinic) initiation and delivery of ART is feasible, acceptable, effective, and address factors related to SDOH such as stigma, lack of time, cost (transportation, food, lost income) to patients of attending ART clinics, and long waiting times [12, 13, 16–18]. There is also growing evidence of the feasibility and acceptability of community-based service delivery models for community-based PrEP (cbPrEP) in low-resource countries [19, 20]. Though some of the elements of international cbPrEP programming will be similar in the US context, it is expected that there may be elements that will need to be addressed for the local context (e.g., racism in the US). Given the high HIV burden in DC and existing SDOH barriers to PrEP uptake, Black residents and low-income neighborhoods are particularly poised to benefit from cbPrEP service delivery models that overcome structural barriers related to SDOH. However, evidence for cbPrEP is limited for the United States (US) context.

While cbPrEP services initiation and continuation are promising, the model has not been tested in the DC area where cbPrEP is not yet standard of practice. To our knowledge, there has only been one published study from a pilot same-day cbPrEP program that provided a 7-day PrEP supply in the Washington, DC area [21]. Of note, several DC-area community-based organizations (CBOs) have been funded to deliver some PrEP and related services, albeit at reduced capacity due to the COVID-19 pandemic. While these services are delivered via CBOs in the communities, they are “community-placed” (i.e., provided out of fixed sites) as opposed to community-based (e.g., at home or via community-based tents). The majority of PrEP initiation currently occurs in healthcare settings, but expansion of PrEP outreach and delivery within other non-healthcare settings (i.e., community-based sites) is part of DOH’s overall strategy to end the HIV epidemic [1].

Our research team has empirical implementation science data demonstrating that community-based ART (cbART) initiation and retention interventions (e.g., at home or via community-based canvas tents) have the potential to overcome structural and societal SDOH barriers in accessing health care, such as by reducing stigma related to uptake of sexual health services [15]. In addition, community-based HIV service delivery interventions have become increasingly used in HIV programming globally and found to improve HIV-related outcomes [15, 17, 18, 22–24]. However, research is needed to inform development and scale up of cbPrEP initiation programs in the DC area, including for Black at-risk adults in the DC area. This is aligned with the *Prevent* pillar of DC Ending the HIV Epidemic Strategies (EHE) strategies. The *DC Ends HIV* plan has a goal of <130 new HIV diagnoses a year by 2030 [5]. To achieve this goal, access to and provision of PrEP is a key priority for DC–as evidenced by the target of 13,000 individuals on PrEP in the *DC Ends HIV* plan [5].

This study aims to adapt a cbART intervention [15] for cbPrEP service delivery for Black adults in the Washington, DC area and will be carried out in two phases. The cbART intervention was originally implemented in Tanzania for female sex workers (FSWs) [15]. The cbART intervention was designed to facilitate ART initiation and retention by reducing SDOH influencing access to ART, including stigma, discrimination, long distance travel, and costs to clients for monthly clinic visits [25]. Specifically, clinical staff were recruited to form a community-based health services team (i.e. one clinician, two nurses, and three peer educators) who provided ART services through the Community-based HIV Testing and Counseling Plus (CBHTC+) mobile or home-based platform. The clinical team provided ART adherence counseling and a one-month supply of anti-retroviral drugs (ARVs) following the government of Tanzania’s test and treat policy. After the first month, clients were given a 2-month ARV supply [25]. Subsequently, stable participants were given a 3-month supply of ARVs per visit [25].

Clients who participated in the cbART intervention were more likely to have initiated ART and less likely to have stopped taking ART for more than 30 days continuously in comparison to clients that participated in the standard facility-based ART delivery structure [15]. By adapting the cbART intervention for a cbPrEP intervention for Black adults in the DC area we hope to obtain similar promising outcomes. In the first phase, we will use the *Assessment*, *Decision*, *Adaptation*, *Production*, *Topical Experts—Integration*, *Training*, *Testing* (ADAPT-ITT) model [26] and the updated Consolidated Framework for Implementation Research (CFIR) 2.0 [27, 28] to adapt the cbART intervention to the cultural, medical, legal, and social context for PrEP service delivery that for Black adults in the DC area [29]. In the second phase, we will pilot test the adapted cbPrEP intervention for acceptability, feasibility, and appropriateness with Black adults.

## Materials and methods

### Setting and study design

The study will take place in Washington, DC and Prince George’s County, Maryland, an adjacent suburban of Washington, DC. Washington, DC is an epicenter of the HIV epidemic in the U.S., and specifically among Black men and women. Additionally, Prince George’s County is the wealthiest Black community in the US, but despite the many socio-structural assets in the county, Prince George’s County has the second highest HIV prevalence and incidence in the state of Maryland. This study will encompass both Washington, DC, and Prince George’s County (hereon referred to as the DC area). Further, *Us Helping Us*, *People Into Living*, *Inc*. (Us Helping Us), our community-based organization (CBO) partner for the proposed study has been providing innovative care and services to improve the health and well-being of Black, gay men who walk through their door in order to reduce the impact of HIV/AIDS in the entire Black community in the DC area. This study will build upon Us Helping Us’ previous studies and high-impact HIV prevention efforts serving the Black community in the DC area that have existed for over a decade, which will save resources by using its existing platforms. Our partnership with Us Helping Us will offer a platform and aid in developing a real-world context into which the proposed intervention can be easily an immediately integrated into existing clinical (including PrEP services) and community-based services of Us Helping Us and other CBOs in the area. Approval for this study was provided by The George Washington University Institutional Review Board (IRB), under reference number: 024120. Consent will be obtained from human study participants.

### Phase I

This phase consists of the Assessment and Decision steps of the ADAPT-ITT model. We will utilize formative qualitative research methods to adapt the cbART initiation intervention [15] for cbPrEP service delivery among Black adults in the DC area.

### Procedures

In the ***Assessment*** phase, we will conduct key informant interviews (KIs) with local CBO leaders and program managers (n = 10), DC and Prince George’s County DOH representatives leading PrEP programs (n = 8), and healthcare providers who provide PrEP services in the DC area (n = 10). In-depth interviews (IDIs) will be conducted with current (n = 12), and potential PrEP users (n = 12) of different sub-groups (Black MSM, Black heterosexual women and men). The interview guides will be informed by the CFIR and will allow us to gain an initial understanding of barriers and facilitators to cbPrEP implementation for different sub-populations of Black adults in the DC area and what complementary tools and activities (e.g., demand generation activities, educational campaign/materials, stigma reduction messaging) for PrEP may support community needs. Findings from the assessment process will inform the Decision phase of ADAPT-ITT, where the research team will decide how to best build upon, adapt, and/or complement the community-based ART (cbART) delivery intervention originally conducted in Tanzania for a cbPrEP intervention for Black adults in the DC area [15].

**Fig 1:**
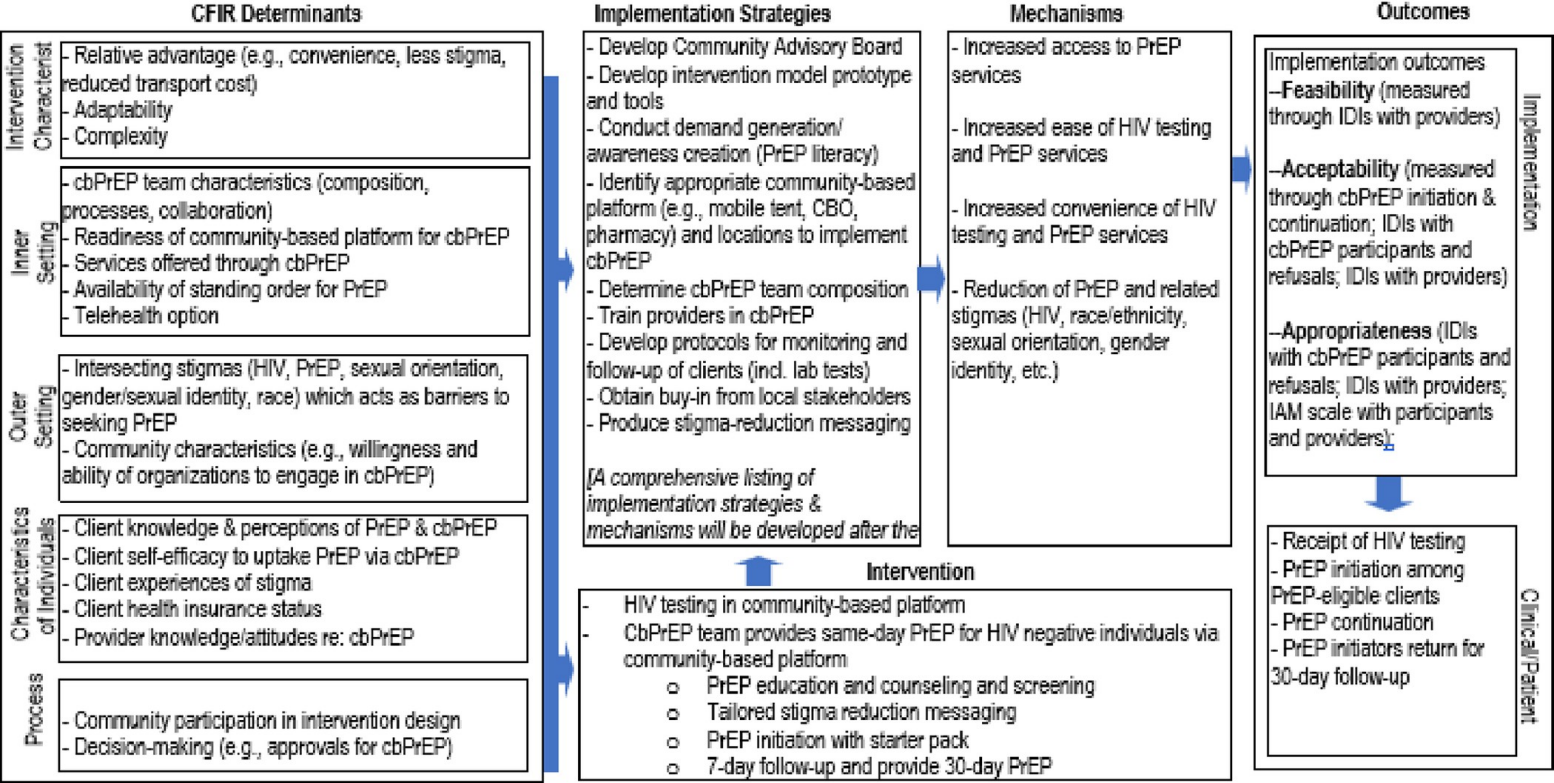
Implementation research logic model for proposed community-based PrEP delivery intervention.

This phase will also include additional literature review of other cbPrEP interventions that have been developed for other populations, particularly in the US setting, that may be relevant and responsive to results from the formative assessment. This step will involve a 1-day workshop with the research team and the CAB to review the results of the formative assessment, taken together with the literature review, with an eye towards developing the cbPrEP intervention. Specifically, workshop participants will be asked to work in groups to provide specific recommendations (based on formative findings) for the following categories of strategies from logic model (Fig 1): (1) Demand generation /Awareness creation; (2) Identification of potential PrEP users (e.g., key locations); (3) Identification of community-based platforms; (4) Team composition and human resource availability and training (depending on who can prescribe); (5) Stigma reduction messaging; (6) Monitoring and follow-up of clients (including laboratory tests); (7) Monitoring/Documentation and reporting; (8) Commodity management procedures (ordering/handling, reporting); and (9) Stakeholder support/buy-in.

Following the ADAPT-ITT framework, as part of the ***Adaptation*** phase, we will invite providers and potential PrEP users back for a “theater test” of potential curriculum, materials, and tools. In these sessions, we will conduct a run-through of the cbPrEP intervention separately with intended target audiences (providers [N = 8] and current and potential PrEP users [N = 10]). We will hold a group discussion with open-ended questions to gather initial reactions; the discussion will be guided by the seven domains of the Theoretical Framework of Acceptability [30, 31], specifically affective attitude, perceived burden, perceived effectiveness, ethicality, intervention coherence, opportunity costs, and self-efficacy. Based on theater testing feedback, as part of the ***Production*** phase, we will develop an initial prototype of the components for the cbPrEP service delivery model including a manual of operations, based on formative assessment findings and the DC DOH guidelines for PrEP services. We will then have ***Topical Experts***, including scientific and Community Advisory Board members, review all materials for further input of the adapted cbPrEP intervention model. These consultations will inform a new draft of the protocol for the adapted cbPrEP intervention.

#### Participant recruitment–Phase I

PrEP providers will be purposely selected from among DC DOH’s Health and Wellness Center, and CBOs providing PrEP, and diversified by affiliation and professional cadre (doctor, nurse practitioner, physician assistant, pharmacist, counselor). CBO staff will be purposely selected based on having knowledge of PrEP programming in the DC area and diversified by CBO affiliation. Potential PrEP users will be recruited from among those testing negative at DC DOH’s Health and Wellness Center. Current PrEP users will also be selected from these two entities; we will diversify based on whether they have missed recent follow-up visits as well as duration of PrEP use.

#### Data analysis

The IDIs and KIs will be transcribed, and two members of the research team will conduct thematic content analysis. Initially, each researcher will read the transcripts independently identifying preliminary codes and subthemes using both inductive and deductive approaches (open coding and coding of theoretical constructs). The focus of the analysis will be to identify how the key determinants identified in the logic model act as facilitators and barriers for cbPrEP, with a lens towards tailoring the cbPrEP intervention for the different sub-groups of Black adults in the DC area. The group sessions from the “theater test” will be recorded and transcribed. The transcripts will be analyzed and coded based on the theoretical constructs Theoretical Framework of Acceptability.

### Phase II

The second phase of the study will take place during the second year of the study and will focus on the ***Integration*, *Training*,** and ***Testing*** phases of the ADAPT-ITT framework. We will pilot the intervention to examine its acceptability, feasibility, and appropriateness of the intervention among 60 Black adult participants, including but not limited to MSM, heterosexual men and women. The intervention will be implemented through existing community-based platforms of Us Helping Us, DC and Prince’s George County DOH, and potentially other community-based platforms (e.g., needle/syringe exchange program) implemented by other local organizations (based on Aim 1 findings). We will conduct a behavioral survey at baseline and around 45 days after baseline; the timing of the follow-up survey will depend on the PrEP follow-up schedule protocol adopted for the intervention. Feasibility, acceptability, and appropriateness will also be assessed among cbPrEP providers.

#### Intervention components

Informed by the findings from phase I, the basic components of the cbPrEP intervention will include the provision of PrEP in a community setting, demand creation strategies, PrEP educational tools/strategies, and stigma reduction messaging. The intervention educational materials and messaging will be tailored to the different subgroups based on findings from phase I. The adapted cbPrEP services curriculum and protocol will be designed to provide social support and facilitate access to PrEP-related services, as well as to reduce PrEP stigma, build self-efficacy, increase PrEP literacy, and assist with navigation around barriers related to SDOH to successful PrEP initiation and use. Additional components and implementation strategies suggested during phase I will be incorporated in the intervention.

#### Participant recruitment–Phase II

The study will be advertised through Us Helping Us, DC Health and Wellness Center, Prince George’s County HIV services, and other local HIV CBOs serving Black adult clients, particularly in the high prevalence wards of Wards 5, 6, 7 and 8 in DC, as well as their social media platforms. The recruitment of study participants will occur through the community-based testing sites (mobile van, tent, or other sites suggested in phase I) of the cbPrEP intervention. All participants who test for HIV will complete a Behavioral Risk Assessment as part of the HIV testing services. Upon testing HIV negative, participants will be asked if they are interested in this study. If interested, they will be screened for eligibility (which includes PrEP eligibility screening), and if eligible, they will be asked to participate in the study and cbPrEP.

Only those who agree to initiate cbPrEP will be eligible to participate in this component of the study. Those who are PrEP eligible, but do not wish to enroll in cbPrEP will be referred to the standard of care, which is the PrEP services offered through fixed sites offered in the DC area, including those offered by Us Helping Us and DC and Prince George’s County HIV services. We will enroll 60 participants. Sample sizes for pilot and feasibility trials are typically around a median of 30 participants [32] thus, a sample size of 60 will allow us to take a phased approach to pilot testing whereby we can refine the intervention (if needed) after the enrollment of groups of 20 participants. Assuming a 30-day PrEP continuation estimate of 70%, a sample of 60 persons would be accurate to within 5 percentage points (with 95% confidence).

Participants will be included if they self-report (1) age of 18 years or above; (2) Black/African American race; (3) tested HIV negative; (4) residence in DC or Prince George’s County; (5) PrEP use (naïve, discontinued); (6) eligible for PrEP per the DC PrEP clinical guidelines and agree to initiate through cbPrEP. Individuals will be considered ineligible if they report: (1) being unable to speak or read English; (2) reside outside of the defined DC/Prince George’s County area; (3) being unable or unwilling to provide written informed consent; (4) being unable to comply with the requirements of the protocol (e.g. persons with mental health conditions, persons who are intoxicated or incoherent for other reasons); or (5) being a member of other vulnerable populations: pregnant women, children or wards of the state.

#### Survey procedures and outcomes

At baseline, participants will complete a self-administered electronic survey in tablets. The survey will elicit information on sociodemographic characteristics, sexual/ gender identity, risk behaviors, PrEP knowledge, attitudes, and self-efficacy, PrEP use history, and the outcomes in **Table 1**. Primary outcomes from the survey with participants include acceptability (as measured through PrEP initiation, continuation), attitudes towards cbPrEP and appropriateness of cbPrEP using validated scales [33], PrEP initiation and self-reported continuation, PrEP follow-up visit attendance, and PrEP knowledge and self-efficacy. The secondary outcomes are other key intermediate factors along the pathway to get to PrEP use, such as stigma. Previously validated scales will be used where appropriate. The follow-up survey will occur after the end of the full 30-day prescription period after the participant is scheduled to return for the follow-up visit (approximately 45 days). The follow-up electronic survey will be e-mailed to the participant and will occur after the scheduled follow-up visit (regardless of attendance) so as not to influence the follow-up visit attendance.

**Table 1:** Measures and definitions.

	Outcomes	Definition [how measured]
*Primary*	*PrEP initiation*	PrEP eligible individuals who agree to start PrEP through cbPrEP and receive the 7-day starter pack [Risk Assessment Questionnaire]
	*PrEP continuation*	Self-report of having used PrEP 80% of the time over the past 30 days. [FU survey]
	*PrEP follow-up visit attendance*	Self-reported attendance for the PrEP follow-up visit. [FU survey; validate with service data]
	*Acceptability of cbPrEP*	Measured through cbPrEP initiation, continuation, and follow-up attendance IDIs with participants and refusals using Theoretical Framework for Acceptability [30, 31]
	*Appropriateness of cbPrEP*	4-item Intervention Appropriateness Measure (IAM) [FU survey] IDIs with participants
	*Attitudes towards PrEP*	Attitudes toward PrEP Scale [Baseline [BL] and FU survey]
	*PrEP knowledge*	Knowledge of what PrEP is & how it works [BL, FU]
	*PrEP self-efficacy*	8-item scale to assess efficacy related to PrEP adherence PrEP, and payment of PrEP, among others [BL, FU]
*Secondary*	*Internalized stigma related to PrEP*	Adapted from existing PrEP studies that measured internalized stigma [BL, FU]
	*Anticipated stigma related to HIV*, *PrEP and/or homophobia*	Stigma related to HIV measured by HIV-related stigma scale (Stigma Index 2.0; sexual behavior stigma measured using American Men’s Internet Survey (AMIS)-2015 (51) [BL, FU survey]

#### Qualitative interviews

*In-depth interviews with participants and non-participants of cbPrEP*. IDIs will be conducted with: (1) Those who were eligible for PrEP but chose not to enroll in cbPrEP (N = 10); and (2) Those who enrolled in cbPrEP (N = 16; diversified by MSM/heterosexual and returning for their PrEP follow-up visit). Non-participants of cbPrEP will be asked about reasons for not enrolling in cbPrEP. Interviews will be guided by the Theoretical Framework for Acceptability, which will address the domains of: ethicality, affective attitude, experienced burden, opportunity costs, perceived effectiveness, stigma, self-efficacy, and intervention coherence [30, 31]. All cbPrEP providers (approximately 8–10) involved in the cbPrEP delivery intervention will be asked to complete a short quantitative survey with items assessing acceptability (*Acceptability Intervention Measure*), feasibility (*Feasibility Intervention Measure*), and appropriateness (*Intervention Appropriateness Measure*) using validated scales [33]. All providers will also be asked to participate in an IDI to elicit their feedback on the cbPrEP intervention regarding acceptability, feasibility, and appropriateness. The IDI will be guided by the Theoretical Framework for Acceptability [30, 31].

#### Data analysis

Frequencies will be generated for characteristics of those who test HIV negative (from Risk Assessment). We will compare socio-demographic and behavioral characteristics of those who initiate and do not initiate cbPrEP from among those who are negative and eligible for PrEP using chi-square test; logistic regression will be used to examine factors associated with cbPrEP initiation. From among those who initiated PrEP (N = 60), we will examine how factors related to SDOH such as risk behaviors, stigma, PrEP self-efficacy and knowledge, and perceived effectiveness are associated with PrEP continuation and PrEP follow-up visit attendance using chi-square test. Analyses will be stratified by gender identity and sexual orientation. The interviews will be transcribed, and two members of the research team will conduct thematic content analysis. Initially, each researcher will read the transcripts independently identifying preliminary codes and subthemes using both inductive and deductive approaches (open coding and coding of theoretical constructs).

## Discussion

The study will develop a cbPrEP intervention and provide evidence for an intervention that can potentially have a significant impact on curbing new HIV infections in DC area-particularly among underserved groups such as Black residents. Although evidence for community-based ART initiation services have been evaluated and demonstrated to be effective in LMICs for more than a decade [8, 27, 30, 31, 33], little research has focused on translating this evidence into the US context. Findings from US government (e.g. PEPFAR/USAID) supported research in LMICs can inform evidence- and community-based service delivery models for Black adults in low income and HIV high prevalence settings in the US. We expect the following outcomes from this study: 1) Intervention prototype, including SOPs and intervention tools (e.g., job aides, stigma reduction messages, educational materials); 2) Dissemination products (e.g., results slide deck and research brief for both lay, scientific, and programmatic/policy audiences); and 3) Peer-reviewed manuscripts. All of which can support the further development of community-based PrEP interventions. The proposed project will advance the translation of effective community-based HIV services using findings from innovative studies conducted in LMICs [8].

A strength of this proposed study is the active collaboration between NGOs/CBOs, universities/research groups, and government (specifically, members of the DC-CFAR (Us Helping Us, DC-DOH, George Washington University Milken Institute School of Public Health) and Population Council) who have met regularly to communicate and collaborate on all aspects of the proposed project. Additionally. Population Council has historically worked internationally but is currently engaging in this project to curb the local epidemic. We will hold regular project meetings via Zoom to ensure stakeholders are updated and supportive of all aspects of the research and intervention implementation. The proposed project will provide evidence for an intervention that can potentially have a significant impact on curbing new HIV infections in DC. We expect that the proposed activities will provide evidence for how a community-based model for PrEP can be implemented in a culturally- appropriate, acceptable, and feasible way for Black adults in the DC area and increase PrEP access by addressing social and structural determinants of health. Through this implementation science research, we will gain insights into which strategies and mechanisms are salient to reaching the goal of improving PrEP access for the target population.
